# The Price of the Publican

**Published:** 1895-04-06

**Authors:** 


### Editor's Letter-Box.

[Our correspondents are reminded that prolixity is a great bar to publication, and that brevity of style and conciseness of statement greatly facilitate early insertion.]

### THE PRICE OF THE PUBLICAN.

Critic writes: Will you permit me to comment on an article entitled "The Price of the Publican," which appeared in a recent issue of The Hospital? In many ways—apart from the question of amount—you recognise the right of the publican. May I express my opinion that you might have gone further? You say the publican does not pay for his license, that of course is incorrect. It may be said the fee is too low, but that is another question. Moreover, the State has formerly recognised claims which, in words once used by Mr. Gladstone, "have grown up in the shadow of the law," for the death duties are levied on the assumption that a license is a continuing possession. Further taking your own illustration of compensation in cases of street improvements I would lay stress on the fact that in such cases public-houses are taken at their value as licensed premises. The article in question goes on to say, "The 'tied houses' in connection with breweries also have little claim to compensation."

Why? If the premises in which a legal trade is carried on are closed, owing to a change in public opinion, why have the interests affected less claim to consideration because the ownership happens to rest in a company instead of an individual? I confess I fail to see the point of some of your criticisms on the tied-house trade. A manufacturer in any industry may have retail shops under managers. Of course, it is conceivable that in a small village poor goods might be passed off on the public owing to a monopoly, but in other cases competition ensures quality. Indeed, the large breweries have done much to improve beer, and it is quite a mistake to suppose that a publican who is free to buy where he likes necessarily serves the public better. As to the grievance about rating—rent is not a *proof* of "value," and assessment committees have a free hand. In conclusion, I may mention one other point. It is often forgotten that local option must render the tied-house system universal. The licenseholder could not afford to risk all his property being swept away by a chance vote. A brewery company might stand the strain, for a loss in one district would probably be partially covered by an increased output in an adjoining one.

*** The writer of the article, to whom the above letter was sent, replies :—When a hard-hearted editor limits the space at a writer's disposal for an article on an importantand complicated subject to two short columns, and ruthlessly cuts down one's "copy" when it exceeds these bounds, an abruptness of statement is bound to creep in.

A license is primarily a privilege, not a commodity that all can buy. If the licensee pays for it, it is rather in the time, influence, and shoe leather expended on winning the good-will of the justices, than in the small fee that is charged. The licensing fee, like the doctor's registration fee, implies the possession of a right, but so much goes to obtain that right besides the fee, that it can hardly be said to pay for it. If anyone could obtain a license by simply paying the fee, one might reasonably say it was paid for; but so long as there are limitations and preferences, so long as one would-be purchaser of the license receives it and another is refused, I think it would be inaccurate to talk of a publican buying his license as he buys the good-will of his business. I admit, however, that my statement was not strictly accurate, though I think that to speak of a license as a purchasable commodity would be distinctly misleading. In spite of the death duties and any assumptions founded on them, a license is not a continuing possession; it is not heritable nor transferable, and each succeeding holder of a licensed business has to obtain his license for himself.

With regard to the "tied house" question, I may say that I object to tied houses as I object to "trusts," "syndicates," "rings," and monopolies in general. To describe the tied houses as merely the retail shop of the brewery is misleading. A wholesale grocer may have as many branch establishments as he chooses, but a retail grocer may open his shop next door at his own pleasure and risk. The law does not decide how many grocers' or drapers' shops we shall have; it is a matter of free competition, and generally the man who gives the best value wins. But the law does settle how many licensed houses there shall be in a given locality, and therefore competition is excluded or, at least, limited to a considerable extent. That the big breweries supply good beer is a strong point in favour of the houses under their control. That I would be one of the first to admit, for I am strongly of opinion that it is not alcohol *per se*, but adulterated alcohol, that is so productive of drunkenness and the diseases and crimes to which drunkenness gives rise. But all brewers are not Guinness or Bass, whose name is a guarantee of excellence. Breweries that make bad liquor have also tied houses, and poison the community, with great profit to themselves. That local option would make tied houses universal does not at all commend them to me; I am not in favour of local option. But my chief objections to tied houses were, I think, clearly stated in my article. The first is that a brewery company, having wealth and influence, can often obtain a licence when it would be refused to a private trader. Nay, I have known of justices withdrawing a license from a publican, not because his house was ill-conducted, but because they wished to give one to a brewery company, and yet did not like to increase the number of licenses. But the strongest reason why tied houses are not deserving of large compensation is that in many cases they were bought not as an investment but as a speculation. The purchasers bought them with the intention of having them confiscated, and being lavishly paid by the State for the confiscation. I do not think my "critic" any more than myself will consider that the nation at large should be taxed in order to gratify such hopes as these.

